# Hours of work and on-call weeks preferences of Canadian midwives: relationships with intention to stay in the profession

**DOI:** 10.1186/s12913-022-08287-6

**Published:** 2022-07-26

**Authors:** Isik U. Zeytinoglu, Firat K. Sayin, Elena Neiterman, Farimah HakemZadeh, Johanna Geraci, Jennifer Plenderleith, Derek Lobb

**Affiliations:** 1 DeGroote School of Business, McMaster University, 1280 Main Street West, Hamilton, Ontario L8S 4M4 Canada; 2 Sobey School of Business, Saint Mary’s University, 923 Robie Street, Halifax, Nova Scotia B3H 3C3 Canada; 3 School of Public Health Sciences, University of Waterloo, 200 University Avenue West, Waterloo, Ontario N2L 3G1 Canada; 4 School of Human Resource Management, Faculty of Liberal Arts and Professional Studies, York University, 4700 Keele Street, Toronto, Ontario M3J 1P3 Canada; 5 College of Midwives of Ontario, 21 St Clair Ave E #303, Toronto, Ontario M4T 1L9 Canada; 6 Faculty of Health Sciences, McMaster University, 1280 Main St W, Hamilton, Ontario L8S 4K1 Canada

**Keywords:** Midwives, Retention, Hours, On-call, Intention to stay, Preferences, Experiences

## Abstract

**Background:**

Midwives have long workdays and work many weeks on call. There is a concern that these extended work schedules can negatively affect their intention to stay in the profession.

**Purpose:**

This study provides evidence on Canadian midwives’ preferences for and experiences with policies and guidelines which limit the hours of work and weeks per year preferred to be on call, and examines the relationship between preferences and midwives’ intention to stay in the profession.

**Methods:**

Data come from our 2018 pan-Canadian survey of midwives. Descriptive statistics of 720 midwives’ preferences and experiences are provided. In the correlations followed by the OLS regressions, 596 midwives’ data are used to test the associations between preferences and intention to stay in the profession. STATA (version 15) is used. A thematic analysis of 274 midwives’ responses to the open-ended survey question is conducted to give voice to midwives on what can be done for retention.

**Results:**

Three quarters of the 720 respondents prefer policies and guidelines to limit hours of work in a 24-hour period, though less than half have policies and guidelines on hours of work. More than half prefer to have fewer on-call weeks or never to be on call, less than a third prefer same number of on-call weeks, and only 2% prefer more weeks to be on call. Midwives are currently working on average 33 weeks per year on call. OLS regression analysis shows that ‘met preference’ for hours of work and on-call weeks are positively associated with intention to stay. In responding to the open-ended survey question, midwives recommend limiting the consecutive hours of work and on-call weeks to manageable hours and weeks to retain them in the profession.

**Conclusion:**

Midwives whose preferences are met are the ones intending to stay in the profession. There is, however, a large number of midwives with ‘unmet needs’ preferring to have policies and guidelines to limit the hours but do not have that currently, and would like to work fewer weeks on call than currently. These are the midwives who are not intending to stay in the profession.

**Supplementary Information:**

The online version contains supplementary material available at 10.1186/s12913-022-08287-6.

## Background

Globally, there is a continued demand for midwives to meet population health needs, and an interest in keeping midwives in the labour force [1]. In this context we focus on the employment conditions of midwives in Canada in terms of hours of work and on-call weeks, and provide evidence on these issues and how they can be associated with midwives’ intention to stay in the profession. Extended work schedules such as long hours of work per day and being on call are considered factors related to healthcare professionals’ retention concerns [2, 3]. Research is also pointing out to the adverse effects of health care professionals’ consecutive work hours on patient safety [4, 5]. In Canada, registered midwives are health care professionals providing primary care during pregnancy, labour, birth and the postpartum period [6]. Midwives in Canada often work in pairs or teams providing care on a 24-hour, seven-day-a-week model [6], though some midwives work alone as “solo” providers [7]. Depending on the needs of the clients, and giving the unpredictable nature of birth, the workday of a midwife can extend to over 24 consecutive hours [8–10]. To identify factors shaping midwives’ intention to stay in the profession, a pan-Canadian study was conducted on midwives’ work experiences, preferences, and intention to stay in the profession. The Research Advisory Committee of this project (consisting of midwives and midwifery educators) suggested that although midwives are committed to the care of their clients and to their profession, they are not satisfied with the long consecutive hours of work and on-call weeks expected of them, and some are considering not staying in the profession. In conversations with midwives [11] we heard that policies and guidelines are necessary to limit the consecutive hours of work per day and the number of weeks per year they are expected to be on call. These consultations and conversations were the impetus of including in our survey questions on policies and guidelines which limit the hours of work, and weeks per year preferred to be on call, which became the foundation of this study.

### Purposes and contributions of the study

The purposes of this study are: (1) to provide evidence on Canadian midwives’ preferences for and experiences with policies and guidelines which limit the hours of work and weeks per year preferred to be on call, and (2) to examine the relationships between midwives’ preferences for hours of work and on-call weeks, and intention to stay in the profession. Data for the study come from our 2018 pan-Canadian survey of midwives. Data for the descriptive statistics on policies and guidelines which limit the hours of work and weeks per year preferred to be on call are from 720 respondent midwives, and data for the OLS regression analysis includes 596 respondents. For the qualitative analysis, responses from 274 midwives to an open-ended survey question are used.

This study contributes to academic and practitioner knowledge by providing evidence on midwives’ long hours of work and on-call weeks, and their preferences for having policies and guidelines to limit continuous hours of work, and preferences for fewer weeks to be on call. The study also provides evidence to policy-makers and decision-makers that those midwives whose preferences are met are the ones who intend to stay in the profession. Midwives with unmet preferences, that is those whose preferences and work experiences are not the same, are the ones who will most likely not stay in the profession. Our pan-Canadian midwifery survey results should be considered evidence for health human resources policy-makers and decision-makers, not only in Canada but also globally, to take appropriate actions to retain midwives in the profession.

### How midwives work in Canada

In Canada, midwives as professionals emerged with the regulation of midwifery in the province of Ontario in 1994. Some provinces (British Columbia, Alberta, Quebec) joined Ontario in the 1990s, while other provinces and territories followed much later [12], and one (Prince Edward Island) is in the process of regulation [13]. Health care nationally is publicly funded so clients are free to select the provider of choice. At present there are too few midwives in Canada to meet the demand for their services. Many family doctors do not provide obstetrical care anymore and thus, the demand is covered by the midwives and obstetrician/gynaecologists. In 2018, the year of our survey, midwives performed 11% of deliveries in Canada, and forced by sheer volume of demand for their services and their small numbers, many, probably upwards of 50% of birthing parents, are turned away because the midwifery practice is full [12].. Since health care provision is a domain of provincial and territorial governments, the way midwifery is practiced across Canada is not entirely uniform. That is, in some provinces and territories, midwives work as employees of local health authorities with set hours of work stipulated by their employment contract, while in others they have the status of an independent contractor who either is directly reimbursed by the government for their services or receives remuneration via membership within a designated clinical practice or, in Ontario, their fees are released to the practice that pays them [14]. Similarly, depending on the province/territory, midwives can work in unionized or non-unionized environments, and can have access to employment benefits (e.g. maternity and parental benefits, sick leave) or lack such services. In some cases, midwives can choose their working arrangements (e.g. working as a solo midwife or join a clinic/team of midwives) and sometimes the choice is not possible. Despite these variations, midwives practicing across Canada have a very uniform model of midwifery practice, which emphasizes being a continuity of care provider and focuses on the provision of care for “normal” (e.g. without medical complication) prenatal, intrapartum and postpartum care for their clients [12]. This means that once a client is in labour, a midwife might be expected to stay with the client for the duration of birth and follow-up care, regardless of how long this process might take. Given the unpredictability of birth and shortages of midwives across Canada, some midwives may be expected to work more hours than stipulated in their contracts (where those are offered).

For hours of work and on-call weeks, midwives’ employment status and compensation are relevant issues. Midwives’ hours of work and on-call weeks are directly affected by their employment status (of an employee or a self-employed midwife), which in turn, affects their entitlement to employment standards legislative coverage (for employees) or exclusion (for self-employed, i.e., independent contractors) [15]. Hours of work and on-call weeks are directly affected by the compensation model they are working in [14], and whether they can be paid through a billable-course-of-care, salary/hourly, a combination of these two arrangements, a contract, or through private fees [15]. In the billable-course-of-care [16] the payment is offered either directly, by health services of the respective provinces, or indirectly, through transfer payment agencies [17]. Midwives can also be remunerated via a monthly salary or in a fixed hourly pay per week [18]. When midwives provide occasional care in hospitals or use their midwifery training to improve access to care or other needs of the communities, there can be salary or fee-for-service funding to compensate work that falls outside the definition of a billable-course-of-care [19]. Midwives paid through private fees receive direct payment for a billable-course-of-care from their clients [15]. Therefore, depending on the compensation model employed in, midwives across Canada may have different ways in which they are reimbursed (or not reimbursed) for the amount of time they spend caring for their clients.

### Theoretical foundation, empirical knowledge, model and hypotheses

Intention to stay is an attitude showing an individual’s attachment to their profession [20] and their interest to continue to work in the profession [21]. In this study we use Steel and Lounsbury’s integrative theoretical model of the retention and turnover process and focus on the intention to stay aspect of the model applying it to the health human resources system of midwives’ employment [22]. As the model states, aspects of the job can affect perceived rewards of staying versus costs of leaving, leading to the ultimate decision of staying or leaving the profession [22].

Empirical research on midwives in Canada presents above discussed various employment models, practice organizations, and compensation [14–19], the need for midwives’ integration into the healthcare system [23], burnout and occupational stress affecting midwives’ intention to leave the profession [2], and the alignment of actual and preferred employment policies contributing to Canadian midwives’ job satisfaction and intention to stay in the profession [15]. In other empirical studies elsewhere, long hours of work and on-call week are found to be significant factors in midwives’ intention to leave the profession [9, 24, 25]. Timely compensation of overtime hours can contribute to intention to stay for midwives working in Swiss maternity hospitals [26]. In allied and other health care professions, for example, for nurses in Canada [27] and managers in Switzerland [28], preferences for shifts, hours of work and on-call work are key issues influencing individuals’ decision to stay in the profession. Having sufficient human resources, in Sweden, where midwives feel that they can manage the demands of the work [29] and ‘greater sensitivity to midwives’ priorities’ in employment in Australia [30 , p.^8^] are shown to contribute to the retention of midwives in the profession.

**Fig. 1:**
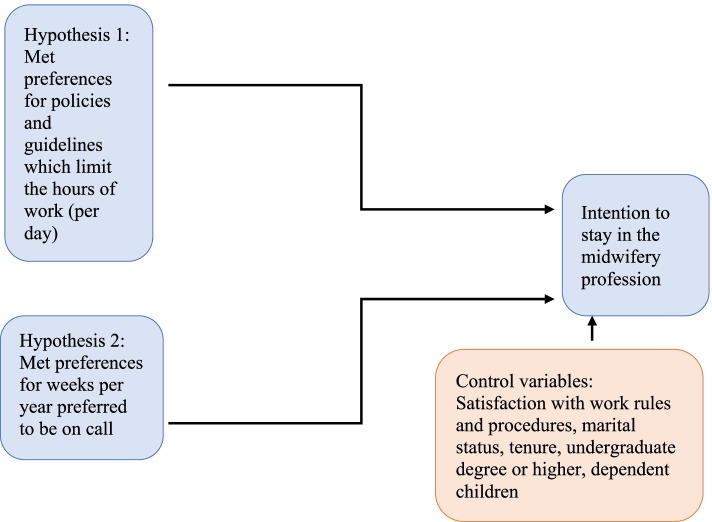
The conceptual model of the relationships between met preferences and midwives’ intention to stay

Using the integrative theoretical model of the retention process [22], and the knowledge gained from the empirical literature on midwives’ work and intention to stay in the profession [2, 9, 14, 15, 24–30] we develop the conceptual model of this study for Canadian midwives, and test the relationships between ‘met preferences’ for policies and guidelines which limit the hours of work and weeks per year preferred to be on call, and intention to stay in the profession (see Fig. 1). The exact wording for ‘hours of work’ used in our 2018 pan-Canadian survey is ‘prefer to have policies and guidelines to limit the number of consecutive hours worked in a 24-hour period’ and ‘currently have policies and guidelines to limit the number of consecutive hours worked in a 24-hour period’. However, for ease of following our conceptualization and arguments in this paper, we also use the shorter wording of ‘hours of work’ hereafter. The wording for ‘on-call weeks’ is written in the survey as ‘preferred weeks per year on call’, and hereafter it is also called ‘on-call weeks’ or ‘being on call’.

In this conceptual model, midwives have ‘met preferences’ if their preferred policies and guidelines which limit the hours of work are the same as their current policies and guidelines of hours of work, and we argue that the midwives with met preferences will intend to stay in the profession. Conversely, midwives who prefer to have policies and guidelines which limit the hours of work but currently do not have such policies and guidelines will have ‘unmet preferences’. We argue that these midwives will not intend to stay in the profession. In addition, those who do not prefer to have a policy and guidelines to limit hours of work but currently work in an environment where such policies and guidelines to limit the hours of work exists will also have ‘unmet preferences’ and, we argue that they will not intend to stay in the profession. Thus, we hypothesize that,

- *Hypothesis 1*. Midwives with ‘met preferences’ for policies and guidelines to limit hours of work will intend to stay in the profession.

The preference for on-call week, whether it is for more, same, or fewer weeks, or never to be on call, will also affect midwives’ intention to stay in the profession. As we present in the conceptual model in Fig. 1, midwives who are working the same number of weeks on call per year as they have currently, will have ‘met preferences’ and will stay in the profession. Conversely, those who prefer to work fewer weeks on call or never want to be on call will have ‘unmet preferences’ and will not intend to stay in the profession. There will be some midwives who prefer more on-call weeks than they currently have, and these midwives will also have ‘unmet preferences’ and will not intend to stay in the profession. Thus,

- *Hypothesis 2.* Midwives with ‘met preferences’ for weeks per year preferred to be on call will intend to stay in the profession.

As presented in the conceptual model (Fig. 1), a number of factors known to affect intention to stay in the profession are included in our study to control for their possible effects on this decision. We control for the effect of satisfaction with work rules and procedures with intention to stay in the midwifery profession, which is a job satisfaction component [31]. Theory on job satisfaction and its effect on intention to stay [32, 33] and the empirical research (see, for example, [31, 34–36]) show the positive association of job satisfaction with intention to stay. Research on midwives also shows job satisfaction as an important factor in their intention to stay in the profession [34, 37, 38] and working hours and work schedules as factors contributing to midwives’ job satisfaction [9, 39]. We believe this job satisfaction component would be better aligned with policies and guidelines which limit the hours of work and weeks per year preferred to be on call, and intention to stay in the profession relationship.

In addition to satisfaction with work rules and procedures, we control for the effects of personal and human capital characteristics that are shown in earlier studies as affecting the intention to stay [40]. The factors we include as control variables are the marital status, tenure on the job, education level, and dependent children. Research on intention to stay shows that being married, having longer tenure, having lower education, and having dependent children, are associated with staying in the organization and the profession [22, 40].

Workers’ voices [41] are important in contextualizing the demands of work, satisfaction with work, and intention to stay. In examining midwives’ preferences for hours of work and on-call weeks, we rely not only on the quantitative data that is tested, but we also listen to Canadian midwives’ voices, share their views, and learn from them what they consider important to retain midwives in the profession.

Findings of our study are discussed within the systems psychodynamic theoretical perspective [42]. This perspective focuses on the societal norms and institutions that influence individuals and theorizes that the societal norms and institutions affect the employees’ behaviour in their organizations. Focusing on the social defense theory of the systems psychodynamic theoretical approach [42] and arguing that when institutional boundaries loosen, the social defense will also weaken or fail, and emotions, with different perspectives and voices will surface. As Petriglieri and Petriglieri (2020: 437) discuss “in these circumstances, a systems psychodynamic lens becomes useful to make sense of shifting relations” [42]. In this environment, as we discuss below, supporting multiple voices is an important practical aim for the workers and their institutions to survive and prosper. Applying the social defense theory to explain midwives’ preferences for policies and guidelines limiting hours of work and preferred on-call weeks per year, we discuss how listening to their voices and recommendations for change in hours of work and on-call weeks can assist policy-makers and decision-makers. Listening to midwives and learning from their experiences can assist policy-makers and decision-makers in implementing changes to enrich the midwifery profession and the healthcare system, and improving the experience of the clients.

## Methods

### Research design

This paper is based on our pan-Canadian survey data collected in 2018 under the, ‘*A new approach to studying retention: following the professional journey of midwives in Canada’* project [hereafter the Midwives Survey] [43]. This project is guided by the Research Advisory Committee consisting of practicing midwives, midwifery educators and organizations, and the project investigators (see Acknowledgement section). This paper is based on the quantitative (questionnaire) and the qualitative (open-ended question) components of the survey. Information on the study and the summary results are further presented elsewhere (see, [44]; Neiterman et. al.: Midwives’ intention to stay in the profession: results of a mixed-methods pan-Canadian study, forthcoming).

### Population, data, and data collection process

The project population is composed of midwives in Canada registered (with full or associate membership) with the Canadian Association of Midwives [45]. The link to the survey was sent to all (the population of) 1690 midwives registered with CAM in 2018. A total of 720 midwives completed the survey (i.e., came to the last page of the online survey) and selected ‘submit the survey’ option. Based on the number of registered midwives in Canada in 2018 when the study was conducted, this yields a 43% response rate. In examining the descriptive statistics of midwives’ preference for and experiences with policies and guidelines which limit the hours of work, and weeks per year preferred to be on call, the data from 720 respondents are used. For the correlations and OLS regression analysis, as explained below, with missing values omitted, there were 596 respondents. For the qualitative component of the survey (open-ended survey question) 274 midwives’ (respondents’) voices on hours of work and on-call weeks are used to learn their views related to these issues and retention of midwives in the profession.

After receiving the ethics approval from the Hamilton Integrated Research Board and research ethics boards of all co-authors’ universities, the data collection process started (see Ethics approvals and consent to participate section below).

Pilot testing of the survey was conducted at the 16th Annual Conference of Canadian Association of Midwives in Victoria, B.C. in 2016, and some wording was slightly modified based on the feedback. We used several recruitment methods to include as many as possible registered midwives across Canada. With the assistance of the Canadian Association of Midwives (CAM) and the Canadian Midwifery Regulators Council (CMRC) we sent email invitations to midwives containing a link to the survey. We also recruited midwives by sharing the project website and study information at midwifery conferences, and welcomed the help of the midwives who offered to share the project link with others. The consent question and self-generated identification code were the only required responses, and the respondents could skip any questions they wanted to. The respondents had to ‘click on submit the survey’ button at the end of the survey for us to collect the data. Respondents were invited to voluntarily enter a draw for three $50 e-gift cards as a token of appreciation for their time; and they had to go to a separate section of the survey not associated with responses to indicate their interest or not. E-mail addresses collected for this draw were separate from the survey data and only accessible to the research coordinator.

### Instrument and measures

The instrument of this paper is the self-completion ‘Midwives Survey’ put together by the project team using their own questions, and theirs’ and others’ published scales (explained below, see also [44]).

Measures for the quantitative (questionnaire) component of the survey include dependent, independent and control variables. Intention to stay in the profession is the dependent variable of this study. It is measured with Lyons’ (1971) three-items scale [21]. Participants are asked to select their level of agreement with each of the items on a five-point Likert scale from 1 = strongly disagree to 5 = strongly agree: (1) If I were completely free to choose, I would prefer to keep working as a midwife; (2) I would like to stay in this profession for a long time; (3) If I had to quit work for a while (for example because of personal/family reasons), I would return to this profession.” The intention to stay variable is created by summing the responses to all three items. The measurement has high reliability (Cronbach’s alpha = 0.89).

The first independent variable is ‘met preference’ for policies and guidelines which limit the hours of work; that is, midwives’ preference for policies and guidelines which limit the hours of work and currently working with policies and guidelines which limit the hours of work are aligned. It is created from the following items: “I would prefer to have policies and guidelines which limit the number of consecutive hours you can work in a 24-hour period:” with response options provided in the survey are on a 5-point Likert scale from “Strongly disagree”, …, to “Strongly agree”, and “Are there policies/guidelines which limit the number of consecutive hours you can work in a 24 hour period?” with response options provided in the survey are “Yes” or “No”. The analysis section below explains how these two questions are used to create an independent variable of ‘met preference’ for hours of work.

The second independent variable is ‘met preference’ for on-call weeks. It is based on the survey question: “How many weeks per year would you prefer to be on-call?”. The response options are “more weeks than I am currently”, “fewer weeks than I am currently”, “the same number of weeks as currently”, and “Never”. In developing this survey question, ‘never’ is included as a possible response option to acknowledge that while some midwives might prefer to have fewer on-call weeks than currently, there might be others who prefer ‘never’ to be on call. Thus, conceptually, while ‘fewer’ can be as low as 1 week per year to be on call, ‘never’ can be only zero weeks on call. It is also important to note that, currently, if a midwife is working they will have on-call weeks. However, when we ask their preference, midwives can say they ‘never’ want to be on call, implying that different applications of midwives’ skills, i.e. employment possibilities, should be considered other than only assisting birthing parents. In the response to the open-ended question, some midwives suggested possibilities to consider. (For more on this, see ‘Responses to open-ended survey question’ section, comments by midwives, particularly in reference to age and having dependent children.) In addition, to learn midwives’ current experiences with on-call work the survey asks: “On average, how many weeks per year you work on-call?” with answers provided used in the descriptive analysis to show midwives’ on-call work experience. Below, additional information is provided to further explain how these variables are used in the analysis.

The control variables are: satisfaction with work rules and procedures, marital status, tenure, education level, and dependent children. For capturing “satisfaction with work rules and procedures”, we use Spector’s (1997) component consisting of 4-items [31]. The following are sample items from the subscale: ‘My effort to do a good job is seldom blocked by red tape,” and “I have too much to do at work” (reversed). Participants are asked to select their level of agreement with each item on a five-point Likert scale from 1=strongly disagree to 5=strongly agree. The satisfaction variable is the sum of the respondents’ answers on each of the four-items. The analysis showed that this measurement is reliable (Cronbach’s alpha = 0.63). Marital status is coded as 0 = single/widowed/divorced/separated and 1 = married/common law. Tenure is included to measure work experience as a midwife and had three categories: 3 years or less (reference), 4 to 10 years, and more than 11 years. Education level is a dichotomous variable where 1 = undergraduate degree or higher and 0 = no undergraduate degree. The dependent children variable is coded as 1 = has dependent children and 0 = has no dependent children.

Measure for the qualitative (open-ended question) component of the survey includes the question “what should be done to help retain midwives in the profession?” This question was placed at the end of the survey and respondents were invited to write their views. The responses are collected and analyzed as the qualitative data. In their responses to this question, participants offered suggestions on what should be done to retain Canadian midwives in the profession. Comments specifically related to the hours of work and on-call weeks are provided in this paper.

### Analysis

For the quantitative (questionnaire) data, STATA 15 is used for the analysis. First, using data from 720 respondents, preferences for and experiences with policies and guidelines limiting hours of work, and weeks per year preferred to be on call and the experience of working on call are analyzed. Second, after creating ‘met preference’ for policies and guidelines limiting hours of work and ‘met preference’ for on-call weeks per year, and including all control variables to be used here, correlations and OLS regression analysis are conducted to test the associations between preferences and intention to stay. In the OLS regression analysis, the observed data with missing values omitted gives us the sample size of 596. We conduct multiple imputation by chained equations (MICE) and find that our OLS regression analysis results are very similar to the results after listwise deletion in terms of significance, direction, and magnitude. The imputed results can be seen in Appendix A. Therefore, we present the unimputed results (i.e., *n* = 596).

In the survey, the response items for ‘the preference to have policies and guidelines which limit hours to work’ are provided on a 5-point Likert scale from “Strongly disagree”, …, to “Strongly agree”, and in the survey the experience of having, that is, ‘currently working with policies/guidelines which limit the hours of work’ is provided with a “Yes” or “No” response option. When creating the ‘met preference’ variable for policies and guidelines which limit the hours of work, we had to decide how to align the response options in the two questions. Thus, we decided on the following in creating this variable. The ‘met preference’ variables are created as follows: “I would prefer to have policies and guidelines which limit the number of consecutive hours I can work in a 24 hour period” are recoded, to align with the experience question below, as Yes (strongly agree and agree) = 1, and No (strongly disagree and disagree) = 0. There were 96 neither disagree or nor agree, and following the median split approach half are coded as “1” (Yes) and half are coded as ‘0’. After recoding this variable, we use another variable, ‘Are there policies and guidelines which limit the number of consecutive hours you can work in a 24 hour period?’ (coded as Yes = 1 and No = 0) to create our first independent variable, ‘met preference’ for policies and guidelines which limit hours of work. If a respondent’s preference and the existence of policies and guidelines are the same (e.g., the respondent prefers to have a policy or guidelines and there is an existing policy or guideline regarding the limiting the number of consecutive hours), then we code our independent variable as 1 = met preference. If they are not the same (e.g., the respondent does not prefer to have a policy and guidelines and there is an existing policy or guideline regarding the limiting the number of consecutive hours) we code the variable as 0 = unmet preference.

The second independent variable, ‘met preference’ for on-call is created by recoding “How many weeks per year would you prefer to be on-call?” with response option “the same number of weeks as currently” (=1) “more weeks than I am currently” (=0), “fewer weeks than I am currently” (=0), and “Never” (=0). While the latter three responses (more weeks than I am currently, fewer weeks than I am currently, and never) are conceptually different, they all reflect an ‘unmet preference’, that is what they want is different from what they are currently doing, suggesting a dissatisfaction with the current experience. Thus, they are coded as 0.

OLS regression is used for inferential analysis. Regarding common method bias, we follow the guidelines of Podsakoff, MacKenzie, Lee, and Podsakoff [46] and used a first-order factor with all measures and did not find any significant factor loadings with any items. Using various plots and statistical tests such as Breusch-Pagan test and Cameron-Trivadi’s decomposition, we do not detect any violations of the model assumptions. The results of these tests can be seen in Appendix A. There are no concerns regarding multicollinearity since the strongest variance inflation factor (VIF) among our variables is 1.81 and the mean VIF value is 1.25.

For the qualitative (open-ended question), responses are collected and analyzed using a thematic analysis framework [47]. After the initial reading of written responses, answers pertaining to the concerns regarding hours of work and on-call schedule are identified as a distinct theme during the analysis. They are further explored to identify the preferences of midwives in relation to their working hours and on-call schedule and integrated in this paper. The goal of this analysis is to give midwives’ “the voice” in retention and to give contextual meaning to the quantitative results.

## Results

### Respondents’ preferences and demographic characteristics

In providing the results, we start with the descriptive statistics of the quantitative (questionnaire) component of the survey. Focusing on the 720 respondents, three quarters prefer to have policies and guidelines limiting consecutive hours of work in a 24-hour period, though less than half have such policies and guidelines in their jobs. More than half prefer to have fewer on-call weeks or never to be on-call, and less than a third prefer same number of on-call weeks, with only 2 % preferring more on-call weeks than currently. Midwives responding to the survey are on-call on average 33 weeks per year. These responses are provided in Table 1.

**Table 1:** Descriptive statistics of hours of work and on-call weeks of Canadian midwives (*n* = 720)

Question	% or weeks	# responded
Prefer to have policies & guidelines to limit consecutive hours of work in a 24-hour period		626 (missing removed)
Strongly agree + agree (yes)	75%	472
Neither agree nor disagree	16%	102
Strongly disagree + disagree (no)	8%	52
Experience of having policies & guidelines to limit consecutive hours of work in a 24-hour period		623 (missing removed)
Yes	46%	286
No	54%	337
How many weeks per year would you prefer to be on call?		622 (missing removed)
More weeks than I am currently	2%	15
Fewer weeks than I am currently	49%	355
The same number of weeks as currently	28%	203
Never	7%	49
On average how many weeks per year are you on call?	33 weeks	596

The descriptive statistics of 596 respondents used in the OLS regression analysis are shown in Table 2. The descriptive statistics show that respondents have moderately high intention to stay in their profession. More than half of the respondents’ preference regarding hours of work is met. However, only one third of respondents’ preference regarding on-call weeks per year is met. The respondents experience moderate satisfaction with rules and procedures. More than three-quarters of the respondents are married and 59% have at least one dependent child. Almost 80% have more than 4 years of tenure as a midwife. The vast majority of the respondents hold at least an undergraduate degree.

**Table 2:** Descriptive statistics and correlations between all variables

*Variables*	*M (SD)*	*1*	*2*	*3*	*4*	*5*	*6*	*7*	*8*	*9*	*10*
1. Intention to stay	11.146 (3.195)	*0.890*									
2. ‘Met preference’ for hours of work	0.577 (.494)	**0.187**	–								
3. ‘Met preference’ for on-call weeks per year	.326 (.469)	**0.321**	**0.087**	–							
4. Satisfaction with rules and procedures	10.416 (2.896)	**0.187**	0.074	**0.265**	*0.632*						
5. Marital status	0.774 (0.172)	0.067	−0.009	0.068	**0.097**	–					
6. Tenure (0–3 years)	0.208 (0.406)	0.041	0.054	**0.094**	0.028	−0.029	–				
7. Tenure (4–10 years)	0.421 (0.494)	−0.006	**−0.109**	− 0.070	0.059	*− 0.001*	**−0.437**	–			
8. Tenure (11 years or more)	.371 (.483)	−0.029	0.066	−0.007	**−0.084**	0.025	**−0.394**	**− 0.655**	–		
9. Education level	0.780 (.415)	0.045	0.03	0.014	−0.032	0.003	0.043	0.010	−0.046	–	
10. Dependent children	.592 (.492)	0.026	−0.005	−0.014	− 0.026	**0.277**	**− 0.096**	**0.106**	− 0.028	0.038	–

*Note*: Cronbach’s α values are presented in italics along the diagonal. Correlation coefficients at the .05 or lower level of significance are in bold. *n* = 596

### Correlations for intention to stay in the profession, met preferences and other variables

We continue with examining the questionnaire data for correlations between variables (*n* = 596). The correlations can be seen in Table 2. Among the statistically significant associations, intention to stay has a stronger positive association with ‘met preference’ for weeks per year preferred to be on call than ‘met preference’ for policies and guidelines which limit the hours of work variable. Independent variables have weak positive associations. Satisfaction with rules and procedures has a positive association with intention to stay and ‘met preference’ for policies and guidelines which limit the hours of work, but it does not have a statistically significant association with ‘met preference’ for weeks per year preferred to be on call. All other control variables, that is, marital status, tenure, education level, and dependent children, have no statistically significant associations with intention to stay.

### OLS regression results for met preferences and intention to stay in the profession

Next, we provide the OLS regression results (as shown in Table 3). ‘Met preference’ for policies and guidelines which limit the hours or work has a positive and significant association with intention to stay. Thus, *Hypothesis 1* is supported. ‘Met preference’ for weeks per year preferred to be on call is positively and significantly associated with intention to stay, supporting *Hypothesis 2*. The adjusted R-squared is 0.13, demonstrating that the variables in the model explain 13% of midwives’ intention to stay. Among control variables, satisfaction with rules and procedures has a significant association with intention to stay. The remaining control variables do not have a statistically significant association with intention to stay.

**Table 3:** OLS regression results between intention to stay and all variables

*Variables*	*Intention to stay*
	B (SE)
Constant	8.351 (.623)***
‘Met preference’ for policies and guidelines which limit the hours of work	1.016 (.249)***
‘Met preference’ for weeks per year preferred to be on call	1.900 (.273)***
*Control variables*	
Satisfaction with rules and procedures	0.107 (.044)**
Marital status	.268 (.306)
Tenure (0–3 years)	Reference
Tenure (4–10 years)	.050 (.332)
Tenure (11 years or more)	−.149 (.337)
Education level	.285 (.296)
Dependent children	.135 (.261)
Adj.R^2^	0.13
*n*	596

**Statistically significant at the .01 level

***Statistically significant at the .001 level

What can be done to retain midwives? Focusing on the responses to the open-ended survey question, we present the views of midwives on what can be done with hours of work and on-call weeks to retain midwives in the profession. On “what can be done with hours of work to retain midwives in the profession?”, in total, 91 responses from midwives refer to issues related to hours of work. Among those who respond to this question, almost half (*n* = 36) feel that the hours of work should be shortened. A few respondents suggest that work hours of midwives sometimes extend past the point where they cannot provide good care to their clients. For instance, one participant notes:

*I know myself and several midwives who have been told [by practice partners] to continue working past 24 hours, even 36 hours when it is not safe, or we lose our jobs.* (Participant 0Y51)

Comparing their work to the practice of other health care providers, midwives suggest that the nature of their practice in and of itself creates a situation where the expectation for a midwife is to provide ongoing care to their clients without backup. One of the midwives offers the following comparison to explain this point:

*I know physicians manage solo practices or small group practices but they do not have same challenges as midwives do as far as requirements of providing 24hr 365 day coverage. Their clients can go to a clinic or hospital for care outside their normal hours. There are no mechanisms for that for midwives.* (Participant 0A101)

Evidently, some respondents feel that not only do they work extra hours, but also that this work is a result of external pressures placed on them either by their managers or practice owners or by the organization of work itself, which does not provide an option to transfer care to a fellow professional.

In those provinces where salaried/hourly compensation is used, given the expectation to work more hours than paid, some respondents (*n* = 10) wrote that they should be paid for those additional hours of work, suggesting to “pay them for all hours worked” (Participant 0F40) or offer a “*compensation for hours worked over full time hours, compensation for work outside normal hours, such as holidays*” (Participant 0 M90). Reflecting on how this issue can be addressed, one midwife proposes “*an overhaul of the salary model so that payment reflects our actual work (ie. twentyfour hours a day/seven days a week)*” (Participant 1F50).

“*Family-friendly hours*” (Participant 0 J121) or “*better hours*” (Participant 0G121, Participant 0 J80) are important for midwives (*n* = 9), with some participants suggesting “*longer resting time after attending a birth or having worked overnight*” (Participant 0X30) is needed. One participant (Participant 1X52) notes that she would prefer set/scheduled hours for time off. A midwife writing that she has worked many years, but her body cannot take the ‘*primary call midwife*’ expectations says:

*I am at the end of my career and although highly skilled and knowledgeable I am challenged to work the usual pattern and hours of a practising midwife as currently defined [in my province]*. (Participant 1A51)

Evidently, midwives feel that work hours that do not meet their expectations make their job more challenging and put a considerable physical strain on their bodies.

Establishing regulations limiting the consecutive hours of work to 12 hours in a day is one of the commonly repeated comments (*n* = 11). Commenting on this, and focusing on their own occupational health and safety, one of the respondents writes:

*Make [us] staying awake [at work] for 72 hours [is] illegal. I can’t even begin to tell you how many times I’ve almost fell asleep at the wheel because I’ve done two births and I need to drive 30 minutes out of town to do a post-partum before I can sleep.* (Participant 1D90)

Echoing this experience, one of the survey respondents makes the following call for action:

*Improve the regulations surrounding safe practice. There absolutely needs to be something in place to ensure that Midwives can sleep after being awake for 24 hours!! It is simply not safe.* (Participant 0E110)

The issue of safety, therefore, both in providing adequate care and in reference to one’s own self-care, is one of the key concerns related to the practice of consecutive long work hours. Regulations on hours of work, particularly hours to be set aside for resting between attending a birth or working overnight, are mentioned as an important consideration by the respondents. The calls to “*design the work so that it does not need to be unsafe [due to] too many hours awake*” (Participant 0E80) and to “*introduce policies on safe work hours*” (Participant 0R111), including “*how many days in a row a midwife can be up all night even with sleep relief in the day*” (Participant 1C122) appear as central demands in written responses of the survey participants. For those who have policies and guidelines on hours of work, enforcing them is perceived as a need.

The respondents provide a number of solutions for resolving the challenge of working long, consecutive hours. One midwife sums these solutions as “*offer [ing] alternate models that include shorter hours*.” (Participant 4F21) Others say “[*currently] the hours are irregular, long (often >24 hours awake)”* (Participant 0E90) and “*long hours of work should be limited* (translation from French) (Participant 0 M60). As this respondent suggests, the expectation embedded into the local model of practice to stay with the birthing client for the duration of labour and delivery makes the hours of work for midwives as unpredictable as birth itself.

Some respondents propose that to retain midwives in the profession an option should be provided for working shifts (*n* = 22) rather than focusing on daily hours of work, and there needs to be a count of weekly hours, with a cap on maximum hours allowed for work. One calls for *“shift options! and capped weekly hours*.” (Participant 0Y51), “*Shift work*” (Participant 0 V90, Participant 0X23) and an option “*to work in shifts when possible*” (Participant 0X121), are listed as key suggestions by the respondents. We should note that some midwives have shift work but for many others apparently this is not an option. Shift work is also alluring to another respondent who wrote:

*As an older midwife I would like to be able to control the number of hours in a row that I work. Shift work could be a solution.* (Participant 0E10)

Long hours of work do not only put physical strain on midwives’ bodies, but also impact their ability to manage their family responsibilities. Suggestions for optional childcare services, which can extend to the evening hours, and “*childcare cooperatives for non traditional working hours,*” (Participant 0A90) are proposed as possible solutions. This is echoed by another participant who writes, “*I really like my job [but] childcare support of some kind would be helpful for me.*” (Participant 0 L100).

On the question of “what can be done with on-call weeks to retain midwives in the profession?”, in total, 183 comments are provided in reference to the midwives’ on-call work experiences. The dominant desire shared in these comments is to have fewer weeks per year to be on call, or have an opportunity to opt out of being on call altogether (*n* = 80). Comments such as “*give us enough off call time to also live good lives*” (Participant 0 L90) or “*[being] on call is too hard for a lot of midwives, especially older midwives*” (Participant 0A110) are provided by the respondents to indicate the toll the on-call schedule had on their ability manage their work demands. One midwife writes that her wish is “*not always be on call*” (Participant 0 L51), suggesting that ‘the expectation to be always available for her clients’ has become a constant in her life. Another respondent calls for “*more shared care/call*,” (Participant 0E50) indicating that working with a partner eases off the pressure of being on call for practicing midwives. The concerns with the demanding on-call schedule are exacerbated by the fact that some respondents do not think they are remunerated for this work. One participant (Participant 3 L81) says that, “*midwives need basic rights such as dedicated off call time and shifts/schedules. They need to be paid to be on call*.” Couched in the language of ‘right’, this demand suggests that survey respondents are both, expected to be on call and not always reimbursed for this working arrangement. This response might be the case for midwives who are salaried or hourly, and perhaps does not apply to those paid by the billable-course-of-care.

Family-friendly/ individual’s personal life-friendly on-call weeks are also suggested (*n* = 9). One midwife directly links “*more family friendly call models*” (Participant 1 J70) to retention. Another midwife comments in the following way:

*I believe [the work should] allow midwives to continue to use their skill sets and contribute to the provision of maternity care when their life doesn't allow them to be on-call 24-7 (i.e., young children, aging, disability, injury, health).* ” (Participant 1N12)

Referring to various changes in personal circumstances of midwives, this participant proposes that during some life transition, on-call work might be especially challenging. Concern about balancing on-call work with other demands is also shared by Participant 0Y40, who says there should be “o*ptions for less on-call work at some points during one’s career (e.g. near retirement, with small children, etc).*”

Beyond the recognition that on-call schedules should be optional in some circumstances, the respondents also comment on the adequacy of compensation for on-call weeks (*n* = 26). One midwife points out:

*I think fair financial compensation [is needed to retain midwives in the profession]. I am going to need to hire a live-in nanny or overnight babysitter as my partner is also on call when we have children. Right now I am really worried about how to afford this with our current salaries. If I was compensated better then this would not be stressful and I would be able to continue to practice how I want.* (Participant 0S71)

Based on the comments from this participant, the on-call schedule does not only pose physical demands on midwives, but also adds financial challenges that need to be addressed, especially in relation to the provision of childcare and other types of care arrangements often falling on women due to the gendered nature of these activities. This participant’s voice is echoed by other midwives facing similar challenges. One of them writes:

*I think that the on-call nature of the work means that childcare is extremely expensive. If your partner has a job with any on-call or out of country requirements (or a single parent) there is a huge cost to the on-call babysitting.* (Participant 0J100)

Hence adequate financial remuneration for being on call, which could only be possible if the on-call work is formally recognized, seemed to be a key need to give midwives an opportunity to stay in the profession, especially when their familial obligations intercepted with professional ones.

Different work settings (*n* = 31) are also suggested for reducing on-call weeks demanded of midwives. Given that birth is unpredictable, having a “break” from providing care during birth is suggested as a potential solution for reducing the number of on-call weeks. Reflecting on the possibility of such professional arrangement, one participant proposes:

*Allow us to use our degrees to do other things at different points in our life where being on-call would be too difficult or we just need a break from the pager lifestyle (i.e., work in a clinic and just do prenatal care and not be on-call, work with Public Health to be home visitors, work in the bilirubin clinic at the hospital etc.), be employed at OB office to do their low risk obstetrics along-side them.* (Participant 0G72)

While in some professional settings midwives are able to have alternative arrangements that enable them not to attend birth for some period of time, for others, such as the participant quoted above, this is not an option. Application of professional skills in other types of care, such as consulting or patient education, is also proposed by Participant 1 M10, who commented that “*midwives have so many skills that can be used in many aspects of healthcare*,” and Participant 0A20 who suggests a need for “*creat [ing] various paths for midwives to take (i.e., expanding scope to allow for differing work settings and paid time in off call positions)*.” As such, a number of respondents (*n* = 26) call for introducing flexibility in the type of work done by midwives, allowing to alternate “full-scope” of practice with “*flexible models*” (Participant 1C50) which would require more routine care and can be offered to midwives who are struggling to continue working unpredictable work hours dictated by the unpredictable nature of birth.

## Discussion

### Main findings

Focusing on the hours of work, the study shows that a substantial majority of midwives responding to the survey prefer to have policies and guidelines limiting consecutive hours of work in a 24-hour period. However, only less than half have such policies and guidelines in their jobs. With respect to on-call weeks, more than half prefer it to be fewer weeks or never to be on-call, less than a third prefer the same number of weeks on-call, and only a small number of respondents (2 %) prefer more weeks to be on-call. These findings provide strong evidence for midwives wanting to have limits to the health care system expectations of working long hours and being many weeks on-call. These findings, of midwives’ unmet preferences for policies and guidelines which limit hours of work, and preferring to work fewer on-call weeks, also suggest how much unmet demand there is for midwives among Canadian birthing parents. OLS regression analysis shows that, as hypothesized, when midwives’ preferences are met for hours of work and on-call weeks, they are more likely to intend to stay in the profession. The responses to the open-ended survey question corroborate these findings with midwives recommending to limit the consecutive hours of work in a day to manageable hours such as 12-hour maximum workdays, and reduce the number of on-call weeks per year to retain them in the profession.

Supporting the integrative theoretical model of the retention and turnover process [22], our study finds the strong impact of the hours of work and on-call weeks aspects of midwives’ jobs affecting their perceived rewards of staying in the profession. Results of this study also support other research findings of Canadian midwives, showing various aspects of the job affecting the intention to stay. In particular, our results are in line with studies showing that midwives’ poor work conditions lead to burnout and stress, and these affect the intention to leave [2, 48], and the need for midwives to be better integrated into the Canadian healthcare system [23] for retention. The results of this study also support earlier findings of the importance of the alignment of actual and preferred employment policies [15] and the interplay between psychosocial and macro-level factors in shaping midwives’ work experiences and their intention to stay in the profession [49]. The findings of this study are also in line with research in other jurisdictions showing that reducing long hours of work, or at least compensating for those overtime hours [26], and reducing on-call weeks are the factors affecting midwives’ intention to stay in the profession [9, 24, 25].

### Implications

Practicing midwifery in Canada with its emphasis on continuity of care and the expectation to be available to clients for the whole duration of birth and immediate postpartum care are taking their toll on midwives. They are experiencing the detrimental effects of this constantly demanding workload on their own health and their ability to care for their families. They give all they can to their clients but not much time and energy are left for their families and themselves. They point out the diametric contradiction of their situation that while they are expected to provide family-friendly care to clients, they themselves are expected to disregard the care needed by their children, partners, other family members, and care for themselves. This contradiction, along with the midwives’ preference to work manageable hours and less on call not being heard, are leading them to question why they are staying in the midwifery profession.

Younger midwives are concerned with not being able to care for their children adequately. For those considering of starting their family, there is worry that if they were to have children, they would not be able to manage work and their own children’s care. Thus, they are debating whether to stay in the profession. Older midwives are saying that their bodies cannot cope with the 24/7 and 365-day continuous demands of the job, and suggest shorter hours of work and less on-call work to be available for midwives at the later stages of their career, particularly nearing retirement. They are saying that their preferences for hours of work and on-call weeks should be met for them to stay in the profession. In sharing their voices, midwives are pointing out to the career and personal life stages of individuals saying that the preferences of individuals can change over time and these preferences should be considered and acted upon by the system for them to stay in the profession.

The implications of our findings can be discussed within the systems psychodynamic theoretical perspective [42], and recommendations can be brought for better work environments for midwives, thus retaining them in the profession. The systems psychodynamic theoretical perspective focuses on “the interaction between collective structures, norms, and practices, on the one hand, and the cognitions, motivations, and emotions of members of those collectives, on the other” [42: 413]. It can help researchers investigate the unconscious forces that underpin the less desirable features of the organizations. In our case, the systems psychodynamic theoretical perspective assists us in investigating and evaluating the interactions between the collective structures, norms, and practices of the midwifery profession, and the midwives place in the Canadian health care system on the one hand, and the cognitions, motivations and emotions of individual midwives, on the other, as they relate to their work in terms of hours of work, on-call weeks, and intention to stay.

The individuals and their organizations are living systems with continuous build up and break up and change, and the individuals in the system are influenced by the social system of their organization [42], and, in our case, their profession and the healthcare system. Applying the systems psychodynamic theoretical perspective [42] to our findings, the behaviour of midwives in the system are influenced by their own interests and goals and, at the same time, influenced by the social system of the midwifery profession and the health care system. The individual interests and goals can align with the profession and the system, but can also be in conflict at the same time. The individual midwife strives for stability and continuity while at the same time wants change or, even, separation. It is obvious that a system of working long hours and many weeks on call has been created as the professional norms and expectations that midwives attempt to adhere to, but they do so with personal cost to their own and their family’s health and well-being. While midwives strive to cope with long hours of work and on-call weeks, the long-term solution requires a system-level change. The voices of midwives and their preferences for shorter work hours and less on-call weeks are indications that some revisions and modifications in the work practices might be considered to retain midwives in the profession. As we hear the midwives’ voices and read their responses to the survey, we suggest that the healthcare system and the midwifery profession consider implementing some of these expectations, and perhaps offering midwives more flexibility in working hours and on-call schedule to promote retention.

There are examples from elsewhere and from medical residents in Canada that can be considered for midwives. For example, having sufficient human resources where midwives feel that they can manage the demands of the work [29] can be considered. Showing some sensitivity to midwives’ priorities in employment and accommodating their preferences [30] can contribute to retaining midwives in the profession. Focusing on midwives in Canada and examining the residency duty hours for medical residents can also give some suggestions. Because of the unpredictable progression of many labours, midwives often have been in clinic for hours through the day and then can be attending deliveries at night that extend their workday much longer than 24 hours. Extended work hours of this magnitude have been a studied issue for Canadian medical residents. The National Standing Committee on Residency Duty Hours [50] recommended that residents limit on-call consecutive work hours to 24–26 hours with a call shift frequency of every 4 days. The underlying concern being patient safety and/or resident burnout, an ongoing perturbation that has yet to be conclusively resolved. Regardless, the most recent agreement of the Professional Association of Residents of Ontario and the Council of Academic Teaching Hospitals of Ontario [51] identifies that obstetric and gynaecology residents should be on-call for a maximum of 24 hours and then off until the next working day. Midwives do not have this option. Perhaps mechanisms similar to medical residents can be created for midwives to contribute to their retention.

### Strengths, limitations, and suggestions for future research

This study is the first national-level data collection on midwives’ work lives and intention to stay. Midwives are an important segment of the Canadian health care system providing a sought after care services to birthing parents. The need for [52] and the shortage of maternity care providers are increasing in Canada [16] and globally [1], thus the sustainability of the midwifery workforce is of critical importance for birthing parents’ health care moving forward. There is limited data on a number of factors and their relationships in retaining midwives in the profession. The strength of our study is that we collect national-level data giving evidence on midwives preferences of and experiences with hours of work and on-call weeks and intention to stay relationship. Another strength of this study is that we collect data through a questionnaire and qualitatively through their responses to an open-ended question. An additional strength of our study is the large sample size, which allows us to analyze midwives’ preferences while controlling for possible effects of a number of other factors studied in earlier research. One of the limitations of our study is that our data are cross-sectional. Therefore, the regression results do not indicate causation, but statistically significant associations. Another limitation of our study is that the open-ended question for the qualitative data was placed at the end of the survey, and thus, the respondents might have been influenced by the questions asked prior to that. At the same time, we would like to note that respondents did not have to answer this question in order to complete the survey, and the large number of responses to the open-ended question show the importance of the hours of work and on-call weeks issues for midwives in Canada.

There are some future research directions that can be derived reflecting on the findings. We recommend future studies to build on our study findings and extend it by focusing on issues not covered here and strengthening the limitations discussed above to continue providing evidence on factors that can contribute to midwives’ retention. Midwives are important in the lives of many birthing parents in Canada and elsewhere, and the findings of this study can be tested in other systems to create improved working conditions for midwives.

## Conclusion

This study provides a comprehensive analysis of hours of work and on-call factors that are related to midwives’ intention to stay in the profession. The study shows that midwives prefer policies and guidelines to limit hours of work in a day so that they will not be expected to work long consecutive hours. They also prefer fewer weeks to be on call. Midwives whose preferences are met are the ones intending to stay in the profession. There is, however, a large number of midwives with ‘unmet preferences’ – many would like to have policies and guidelines to limit the hours but do not have that currently, and many would like to work fewer weeks on call than currently working. These are the midwives who are not intending to stay in the profession. Based on the findings, we recommend policy-makers and decision-makers consider midwives’ preferences in terms of hours of work and on-call weeks as these are important factors for retention. While our results are reflecting Canadian midwives’ preferences and experiences providing evidence and highlighting their importance to Canadian decision-makers and policy-makers on actions to take, we recommend applying this knowledge to similar jurisdictions elsewhere to retain these highly skilled health care professionals in the profession.

## Supplementary Information

**Additional file 1: Appendix A.** Further information on the imputation process and model assumption tests.

## Data Availability

The dataset this paper uses is originally collected and owned by Drs. Zeytinoglu, HakemZadeh, Neiterman, and Lobb. The data can be available only after the owners have completed using the data for their submissions (journal articles and other media outputs). The statistical output that this paper is based on can be available for review, from the second author, upon request.
