# Supplementary material for: Hours of work and on-call weeks preferences of Canadian midwives: relationships with intention to stay in the profession
Source: BMC Health Serv Res. 2022 Jul 26;22:950. doi: 10.1186/s12913-022-08287-6 (PMC9316842; doi:10.1186/s12913-022-08287-6)
Supplement: Supplementary file 1 — Additional file 1: Appendix A. Further information on the imputation process and model assumption tests. [file 12913_2022_8287_MOESM1_ESM.docx]

**Hours of work and on-call weeks preferences of Canadian midwives: Relationships with intention to stay in the profession**

**Appendix A**

**Parameterization of the imputation algorithm**

Linear regression, logistic regression, and multinomial logistic regression are used as elementary imputation models. 100 imputations were generated and 10 iterations for the burn-in period for each chain were used. The convergence was established with multiple independent chains. The plausibility of imputed values was checked using subject matter knowledge of the authors. The high level of resemblance of the original results and the imputed values further supports the plausibility of the imputed values.

**TABLE S1. Multiple-imputation based model results**

| Variables | Intention to stay |  |
| --- | --- | --- |
|  |  |  |
|  | B (SE) |  |
| Constant | 8.479 (.618)*** |  |
| ‘Met preference’ for hours of work | 0.984 (.251)*** |  |
| ‘Met preference’ for on-call weeks per year | 1.876 (.271)*** |  |
| *Control variables* |  |  |
| Satisfaction with rules and procedures | 0.105 (.044)** |  |
| Marital status | .173 (.302) |  |
| Tenure (0-3 years) | Reference |  |
| Tenure (4-10 years) | .025 (.331) |  |
| Tenure (11 years or more) | -.111 (.333) |  |
| Education level | .288 (.292) |  |
| Dependent children | .146 (.258) |  |

Note: * Statistically significant at the .05 level; ** at the .01 level; *** at the .001 level.

**TABLE S2. Fraction of missing information (FMI) results**

| Variables | Fraction of missing information (FMI) |  |
| --- | --- | --- |
|  |  |  |
| Constant | 0.010 |  |
| ‘Met preference’ for hours of work | 0.026 |  |
| ‘Met preference’ for on-call weeks per year | 0.016 |  |
| *Control variables* |  |  |
| Satisfaction with rules and procedures | 0.021 |  |
| Marital status | 0.005 |  |
| Tenure (0-3 years) | Reference |  |
| Tenure (4-10 years) | 0.001 |  |
| Tenure (11 years or more) | 0.005 |  |
| Education level | 0.005 |  |
| Dependent children | 0.005 |  |

**Assumption tests**

**White’s test**

H0: Homoskedasticity

Ha: Unrestricted heteroskedasticity

chi2(36) = 55.48

Prob > chi2 = 0.0200

**Cameron & Trivedi's decomposition of IM-test**

| Source | Chi-squared | df | p |
| --- | --- | --- | --- |
| Heteroskedasticity | 55.48 | 36 | 0.02 |
| Skewness | 35.99 | 8 | 0 |
| Kurtosis | 10.76 | 1 | 0.001 |
| Total | 102.23 | 45 | 0 |

**Breusch–Pagan/Cook–Weisberg test for heteroskedasticity**

Assumption: Normal error terms

Variable: Fitted values of in_to_stay

H0: Constant variance

chi2(1) = 23.77

Prob > chi2 = 0.0000
